# Association Between Contraceptive Use and Gestational Diabetes: Missouri Pregnancy Risk Assessment Monitoring System, 2007–2008

**DOI:** 10.5888/pcd11.140059

**Published:** 2014-07-17

**Authors:** Brittney A. Kramer, Jeremy Kintzel, Venkata Garikapaty

**Affiliations:** Author Affiliations: Brittney A. Kramer, Jeremy Kintzel, Missouri Department of Health and Senior Services, Jefferson City, Missouri.

## Abstract

**Introduction:**

The efficacy and safety of contraceptives have been questioned for decades; however, whether a relationship exists between hormonal contraceptives and gestational diabetes (GDM) is undetermined. The aim of this study was to investigate whether maternal risk for GDM was influenced by type of contraceptive method used before pregnancy.

**Methods:**

Data collected in 2007 and 2008 by the Missouri Pregnancy Risk Assessment Monitoring System (PRAMS) were analyzed to determine if type of contraception before pregnancy influenced maternal risk for GDM. We used a logistic regression model to determine the adjusted odds for GDM given exposure to hormonal forms of contraception.

**Results:**

Of the 2,741 women who completed the 2007–2008 PRAMS survey, 8.3% were diagnosed with gestational diabetes, and 17.9% of the respondents had used hormonal contraceptive methods. Women who used hormonal methods of birth control had higher odds for gestational diabetes (adjusted odds ratio [AOR] = 1.43; 95% confidence interval [CI], 1.32–1.55) than did women who used no contraception. A protective effect was also observed for women who had used barrier methods of contraception (AOR = 0.79; 95% CI, 0.72–0.86).

**Conclusion:**

Findings suggest there may be a relationship between type of contraceptive method and GDM. More research is needed to verify contraception as a potential risk factor for GDM.

## Introduction

Over the years, concerns have been raised about the possible association between hormonal contraceptives and various chronic diseases, including cardiovascular disease, breast cancer, and metabolic dysfunction. However, little is known about hormonal contraceptive use and its role in the development of gestational diabetes (GDM). Research has established a relationship between oral, hormonal contraceptive use and increased levels of serum glucose, insulin, and altered lipid profiles (1–4); however, much of the research is still unable to reach consensus on the long-term effects of contraceptives and an increased risk for GDM. Studies have examined the effects of contraception on the metabolisms of women who were previously diagnosed with GDM and found that women using hormonal methods of contraception had a higher risk for type 2 diabetes than did women using nonhormonal contraceptive methods (4). Nevertheless, no studies have investigated the effects of hormonal contraceptive use before pregnancy and the risk for GDM.

GDM is often diagnosed in weeks 24 through 28 of pregnancy and is characterized by no previous history of diabetes (type 1 or type 2) and high blood glucose levels (5). If left uncontrolled, GDM can result in macrosomia (“fat” baby), which can cause complications at birth and potentially force the mother to have a caesarian delivery. Other potential side effects involve preeclampsia and hypoglycemia (low blood glucose) in the mother, jaundice in the baby, and an increased risk for type 2 diabetes in both the mother and child later in life. GDM is estimated to affect 2% to 10% of all pregnancies, although US prevalence has increased since the late 1980s and has more than doubled since 1990 (6,7). Therefore, it is crucial to identify and understand risk factors for GDM to better tailor preventive and intervention measures.

The Missouri Pregnancy Risk Assessment Monitoring System (PRAMS) is an ongoing, population-based survey designed to identify and monitor select maternal experiences, attitudes, and behaviors that occur before, during, and shortly after pregnancy among women delivering a live infant. Data from PRAMS can be used to determine whether a relationship exists between a woman’s contraceptive use before pregnancy and the development of GDM. 

Research conducted by Visser et al compared hormonal and nonhormonal contraceptive use by diabetic women and found that high-dose oral contraceptives impaired glucose homeostasis (8). Likewise, Diab and Zaki found that fasting blood glucose was higher among users of oral contraceptives and Depo-Provera (depot-medroxyprogesterone acetate, DMPA), a progestogen-only reversible hormonal contraceptive injected every 3 months (9). After reviewing these studies, we hypothesized that an increased risk for GDM would be observed among women who had used hormonal methods of contraception. Our objective was to identify whether, after controlling for other factors, women who used hormonal methods of contraceptives before their pregnancy were more or less likely to develop GDM. 

## Methods

### Data collection

PRAMS is conducted by the Missouri Department of Health and Senior Services as part of the national program sponsored by the Centers for Disease Control and Prevention (CDC). PRAMS is administered in 40 states and in New York City and represents approximately 78% of all US live births. Missouri began administering PRAMS in 2007. PRAMS collects information from a sample of women whose names are drawn from recent birth certificates (2 to 4 months after birth). A survey is mailed to each woman with telephone follow-up to those who do not respond. Responses are weighted to represent all live births in a given year. In Missouri, 2,741 women responded to the survey in 2007–2008, for an average weighted response rate of 63.9%.

### Study population

Missouri has participated in PRAMS continuously since 2007, beginning with Phase V of the survey (2007–2008). Missouri is collecting Phase VII data (2012 to present); we used Phase V data because the Phase V questionnaire asked participating women to identify the type of contraceptive they were using prepregnancy and postpartum, if applicable. Women could respond that they or their partner were using tubal ligation, vasectomy, birth control pills, condoms, injections (monthly or every 3 months), contraceptive patches, a diaphragm, a cervical ring, an intrauterine device, the rhythm method, withdrawal, or another method. They could also respond that they were not sexually active.

Women were identified as having been diagnosed with GDM if they reported that they had high blood glucose (diabetes) that started during the current pregnancy or if they had reported GDM on the matched birth certificate and did not respond in PRAMS that they had high blood glucose (diabetes) that started before the current pregnancy. This approach replicates GDM prevalence reported for Missouri by CDC’s PRAMS C-PONDER query tool for 2007; 2008 data are unavailable in C-PONDER because Missouri did not meet the minimum required weighted response rate threshold for inclusion of 70%.

Methods of prepregnancy contraception were categorized as hormonal, barrier, fertility awareness, other, or none. Methods of hormonal contraception included the pill, injections (received monthly or every 3 months), contraceptive patches, cervical ring, or intrauterine devices (IUD). Barrier methods included condoms or diaphragm, and fertility awareness methods included the rhythm or withdrawal methods. Respondents who reported that they were not using birth control when they became pregnant or reported that they were not using any of these specified methods were categorized as not using any contraception. All others were categorized as “other.” These categories were selected on the basis of CDC categorization of birth control methods as either reversible birth control methods or permanent methods of birth control. All permanent methods (tubal ligation or vasectomy) were excluded because conception is next to impossible after these procedures, and the respondents were reporting this method as their contraceptive method before their most recent pregnancy.

### Data analysis

Data were analyzed using SAS Enterprise Guide 4.3 (SAS Institute, Inc, Cary, North Carolina). A regression model was constructed with GDM as the outcome variable and other variables as predictor variables, representing respondents’ demographics and preconception health, pregnancy health, and other birth outcomes. These variables included the mother’s age, education level, race, Medicaid status (at delivery), marital status, contraceptive method, pregnancy intent, prepregnancy body mass index (BMI in kg/m^2^, calculated from participants’ self-reported height and weight), pregnancy weight gain, income, folic acid multivitamin use, adequacy of prenatal care, number of prenatal stressors experienced, use of assisted reproductive technology or fertility drugs, preterm or low birth weight baby, WIC participation, and a range of other prenatal maternal morbidities, including vaginal bleeding, high blood pressure, and preterm labor.

All variables from the univariate model were selected as predictor variables for the final, multivariate model. A backward stepwise selection was performed on all variables, which included respondents’ age, education level, race, Medicaid status, marital status, pregnancy intent, contraceptive method, prepregnancy BMI, income, folic acid multivitamin use, and adequacy of prenatal care. For the adjusted multivariate model, methods of contraception were categorized and analyzed as hormonal, barrier, fertility awareness, other, or none. The final model was tested for collinearity, and all predictor variables were significant at *P* < .001.

## Results

Of the 2,741 women who completed the 2007–2008 PRAMS, 8.3% reported having been diagnosed with GDM in their most recent pregnancy. The most prevalent form of contraception used by Missouri PRAMS respondents was hormonal methods of contraception (17.9%), followed by barrier methods (17.2%), fertility awareness/rhythm method (6.8%), and other (2.3%). Of the total sample, 56% reported using no contraception. 

Results of multivariate logistic regression indicated that women who used hormonal birth control methods were 1.4 times more likely to develop GDM than were those who did not use any birth control method (95% confidence interval [CI], 1.32–1.55, *P* < .001) (Table). Several other variables were associated with higher odds for developing GDM: women aged 30 or older compared with those younger than 20 (AOR = 1.50; 95% CI, 1.34–1.67; *P* < .001), women who received adequate prenatal care compared with women who received inadequate or intermediate prenatal care (AOR = 2.36; 95% CI, 2.16–2.58), women enrolled in Medicaid at delivery compared with women who were not enrolled in Medicaid at delivery (AOR = 2.58; 95% CI, 2.36–2.81), women who identified as nonwhite or nonblack compared with white women (AOR = 5.54; 95% CI, 4.90–6.25), and women who were overweight or obese before pregnancy compared with women with a normal BMI before pregnancy (AOR = 3.04; 95% CI, 2.84–3.24) (Table). A protective effect for GDM was observed for women who had used barrier methods for contraception compared with women who did not use any contraceptive methods (AOR = 0.79; 95% CI, 0.72–0.86) (Table) and for women with an unintended pregnancy compared with women with an intended pregnancy (AOR = 0.39; 95% CI, 0.37–0.42) (Table).

**Table:** Maternal Characteristics and Odds of Developing Gestational Diabetes, Missouri Pregnancy Risk Assessment Monitoring System (N = 2,741), 2007–2008^a^

Characteristic	Prevalence	Adjusted Odds Ratio (95% CI)
**Contraceptive method**		
Hormonal	8.2	1.43 (1.32–1.55)
Barrier	5.9	0.79 (0.72–0.86)
Fertility awareness	10.0	1.38 (1.24–1.53)
Other	12.0	0.59 (0.47–0.73)
None	7.7	1 [Reference]
**Age, y**		
<20	4.9	1 [Reference]
20–29	7.4	0.98 (0.89–1.08)
≥30	11.1	1.50 (1.34–1.67)
**Education level**		
Less than high school	7.2	1 [Reference]
High school or more	8.4	1.38 (1.27–1.50)
**Race**		
White	7.9	1 [Reference]
Black	7.7	1.32 (1.22–1.43)
Other	18.0	5.54 (4.90–6.25)
**Medicaid status at delivery**		
Medicaid	9.0	2.58 (2.36–2.81)
Non-Medicaid	7.4	1 [Reference]
**Pregnancy intent**		
Unintended	6.3	0.39 (0.37–0.42)
Intended	9.6	1 [Reference]
**Prepregnancy weight status (BMI, kg/m^2^)**		
Underweight (<18.5)	2.3	0.79 (0.64–0.97)
Normal weight (18.5–24.9)	4.8	1 [Reference]
Overweight/obese (≥25.0)	11.5	3.04 (2.84–3.24)
**Income, $**		
<15,000	7.3	0.57 (0.51–0.64)
15,000–24,999	9.3	0.73 (0.65–0.82)
25,000–49,999	9.2	0.72 (0.66–0.80)
≥50,000	7.8	1 [Reference]
**Folic acid multivitamin use**		
Use 4 times weekly	8.1	1 [Reference]
No use	8.1	1.26 (1.16–1.37)
**Prenatal care status**		
Adequate/plus	9.3	2.36 (2.16–2.58)
Inadequate/intermediate	4.4	1 [Reference]
**Marital status**		
Married	8.5	0.83 (0.77–0.89)
Not married	7.7	1 [Reference]

Abbreviation: CI, confidence interval; BMI, body mass index.

a All variables listed were included in final, multivariate model and found significant at *P* < .001

## Discussion

This study provides evidence that hormonal contraceptive methods may increase a woman’s risk for GDM in her following pregnancy, even after adjusting for maternal age, race, education and income level, marital status, Medicaid status at delivery, and type of prenatal care received. Other variables that were associated with higher odds for GDM were women who had a high school education or more, were 30 years or older, were of nonwhite or nonblack race, and were overweight or obese before pregnancy. These findings are consistent with those of other studies, one of which found that risk for GDM was correlated with increasing maternal age, high prepregnancy BMI, and high weight gain during pregnancy (10). Similarly, another study found that woman from racial/ethnic minority groups had a higher prevalence of GDM and that age and high BMI were also independent risk factors (11). It has been long accepted in the medical field that higher BMI, increasing age (>25 y), and nonwhite race are risk factors for GDM (12). Our study, along with others, validates this consensus and suggests that intervention strategies should continue to focus on these demographic groups.

Other variables associated with higher odds for GDM included adequate access to prenatal care and being enrolled in Medicaid at delivery. Women who are enrolled in Medicaid and are receiving adequate prenatal care are more likely to see a doctor more frequently during their pregnancy and therefore have any pregnancy complications, including GDM, observed and diagnosed. This observation was also determined by Alexander and Cornely, who found that women who received intensive prenatal care were more likely to have more pregnancy complications, but also the most preferable birth outcomes (13). Furthermore, Griffin and colleagues determined that Medicaid expansion programs allowed women to receive the same treatment and prevention services on par with adequate prenatal care (14). These studies explain the higher odds observed for women who are receiving adequate prenatal care or Medicaid services; they are more likely to be diagnosed with GDM because they are more likely to be receiving frequent adequate care and therefore to be screened and diagnosed with it as opposed to women who may not be able to receive adequate care and consequently miss the opportunity to be diagnosed with GDM.

Protective effects were seen in women who had not intended or planned their pregnancy or who used barrier methods for contraception. As described previously, women who do not plan their pregnancy often underuse prenatal care services and receive inadequate care (15). This effect is the opposite of what was described previously, since women with an unintended pregnancy often inadequately seek and use prenatal care, so they are less likely to be seen and diagnosed by a doctor for GDM. On the basis of our hypothesis, women who are not using any form of hormonal contraception should be at an observed lower risk for developing GDM. Women who practice barrier methods of contraception are not increasing levels of hormones in their bodies and do not have the potential and undetermined side effects and, we propose, have a lower risk for GDM.

PRAMS data are subject to several limitations. First, PRAMS is a self-reported survey administered 2 to 6 months after the birth of a child, and results may be subject to recall bias. Second, weighting may not completely adjust for bias resulting from nonresponse. Third, PRAMS surveys are sent to a sample of women who delivered live babies in Missouri, and data are not generalizable to pregnant women with other outcomes or in different states.

This is the first study to evaluate the relationship between type of contraception used before pregnancy and risk for GDM. However, many studies have determined that hormonal methods of contraception have the potential to alter several homeostatic mechanisms including the hypothalamic pituitary axis as well as cortisol and carbohydrate metabolism. Burke suggested that prolonged exposure to high-dose estrogen treatment was associated with higher levels of unbound cortisol, which implied interference with cortisol homeostasis at the hypothalamic level (16). These higher levels of cortisol contribute to glucose intolerance and insulin resistance, which if left untreated can lead to diabetes (17).

This study’s significant finding correlating contraceptive method and risk for GDM merits further exploration. The prevalence of GDM continues to increase worldwide, and definitive screening and preventive measures are needed to deter its chronic lifelong complications. Although researchers have not established a causal relationship between hormonal contraception use and GDM, results of our study suggest there may be an underlying correlating mechanism. More research is needed to assess hormonal contraception use as a potential risk factor for GDM.
